# Supplementary material for: Comparing Five Generative AI Chatbots’ Answers to LLM-Generated Clinical Questions with Medical Information Scientists’ Evidence Summaries
Source: medRxiv. 2025 Sep 27:2025.09.24.25336199. Preprint. [Version 1] doi: 10.1101/2025.09.24.25336199 (PMC12486027; doi:10.1101/2025.09.24.25336199)
Supplement: Supplement 2 [file media-2.pdf]

Appendix B. List of PICO Questions

Question number	Question	LLM question source	Question category
1	In patients with type 2 diabetes and moderate renal impairment, does the use of SGLT2 inhibitors compared to DPP-4 inhibitors lead to a greater reduction in cardiovascular events?	ChatGPT	Treatment
2	Among pregnant women in their first trimester, how does maternal obesity, compared to normal maternal weight, affect the risk of developing gestational diabetes?	ChatGPT	Etiology
3	In patients diagnosed with early-stage non-small cell lung cancer, how does the presence of lymphovascular invasion on pathology reports compare to its absence in predicting 5-year survival rates?	ChatGPT	Prognosis
4	In patients with acute myocardial infarction, does a percutaneous coronary intervention (PCI) strategy compared to a conservative medical management strategy improve long-term survival?	Google Gemini	Treatment
5	In adult patients with recurrent urinary tract infections (UTIs), does a history of previous antibiotic use increase the risk of developing antibiotic-resistant UTIs?	Google Gemini	Etiology
6	What is the long-term prognosis of a patient with newly diagnosed idiopathic pulmonary fibrosis (IPF) and a forced vital capacity (FVC) of 50% predicted?	Google Gemini	Prognosis
7	In patients with chronic lower back pain, how effective is physical therapy compared to corticosteroid injections in reducing pain and improving mobility?	Microsoft Copilot	Treatment
8	For patients with type 1 diabetes, how does early viral infection exposure compare to no early viral infection in contributing to the onset of the disease?	Microsoft Copilot	Etiology
9	In patients with early-stage non-small cell lung cancer, how does the presence of circulating tumor cells affect overall survival compared to those without detectable circulating tumor cells?	Microsoft Copilot	Prognosis
10	For patients with chronic lower back pain, is physical therapy more effective than non-steroidal anti-inflammatory drugs (NSAIDs) in improving pain and functional mobility over six months?	ChatGPT	Treatment

11	In children under the age of five, does early exposure to secondhand smoke, compared to no exposure, increase the likelihood of developing asthma or other chronic respiratory conditions?	ChatGPT	Etiology
12	In patients with newly diagnosed multiple myeloma, how does the detection of high-risk cytogenetic abnormalities compared to standard-risk cytogenetics affect overall survival over a 3-year period?	ChatGPT	Prognosis
13	For adults with type 2 diabetes mellitus, does the addition of a GLP-1 receptor agonist to standard metformin therapy reduce the risk of major adverse cardiovascular events compared to metformin alone?	Google Gemini	Treatment
14	In children with autism spectrum disorder (ASD), does exposure to environmental toxins during pregnancy or early childhood increase the risk of developing ASD?	Google Gemini	Etiology
15	How does the presence of atrial fibrillation affect the prognosis of patients with acute myocardial infarction?	Google Gemini	Prognosis
16	For adult patients with Type 2 diabetes, does the addition of a GLP-1 receptor agonist to standard metformin therapy improve glycemic control better than adding a DPP-4 inhibitor?	Microsoft Copilot	Treatment
17	Among individuals diagnosed with celiac disease, what role does genetic predisposition play in comparison to environmental factors such as diet and hygiene practices?	Microsoft Copilot	Etiology
18	For individuals diagnosed with chronic kidney disease, what is the impact of elevated serum creatinine levels on the progression to end-stage renal disease compared to those with lower serum creatinine levels?	Microsoft Copilot	Prognosis
19	In patients with newly diagnosed atrial fibrillation, does the initiation of direct oral anticoagulants (DOACs) compared to warfarin reduce the risk of stroke and systemic embolism without increasing major bleeding incidents?	ChatGPT	Treatment
20	For adults with a history of hypertension, how does high dietary sodium intake, compared to a low-sodium diet, influence the incidence of stroke and cardiovascular events?	ChatGPT	Etiology

21	In individuals with chronic hepatitis B infection, how does a persistently elevated ALT level compared to a consistently normal ALT predict the progression to cirrhosis within 5 years?	ChatGPT	Prognosis
22	In patients with metastatic non-small cell lung cancer (NSCLC) and EGFR mutations, does treatment with a tyrosine kinase inhibitor (TKI) compared to chemotherapy improve overall survival and quality of life?	Google Gemini	Treatment
23	In patients with chronic obstructive pulmonary disease (COPD), does exposure to air pollution increase the risk of exacerbations and mortality?	Google Gemini	Etiology
24	What is the risk of cardiovascular events in patients with type 2 diabetes mellitus and a persistent albuminuria level of 30 mg/g?	Google Gemini	Prognosis
25	In patients with acute ischemic stroke, does mechanical thrombectomy within 6 hours of symptom onset lead to better functional outcomes compared to intravenous thrombolysis alone?	Microsoft Copilot	Treatment
26	In elderly patients with Alzheimer's disease, how does chronic exposure to air pollution compare with genetic factors in influencing the development of the disease?	Microsoft Copilot	Etiology
27	In patients with heart failure with reduced ejection fraction, how does the presence of a baseline elevated BNP level affect the risk of hospitalization for heart failure compared to those with lower BNP levels?	Microsoft Copilot	Prognosis
28	For hospitalized patients with severe community-acquired pneumonia, does the addition of corticosteroids to standard antibiotic therapy reduce mortality and the length of hospital stay compared to antibiotics alone?	ChatGPT	Treatment
29	In middle-aged adults, does long-term high alcohol consumption, compared to moderate or no alcohol use, increase the risk of developing liver cirrhosis?	ChatGPT	Etiology
30	For patients who have suffered an initial ischemic stroke, how does the presence of intracranial artery stenosis on imaging compare to no detected stenosis in forecasting the risk of recurrent stroke within 3 years?	ChatGPT	Prognosis
31	In children with asthma, does the addition of inhaled corticosteroids to inhaled beta-agonists reduce the frequency of acute exacerbations and improve lung function compared to inhaled beta-agonists alone?	Google Gemini	Treatment

32	In postmenopausal women, does hormone replacement therapy (HRT) increase the risk of developing breast cancer?	Google Gemini	Etiology
33	What is the likelihood of disease progression in patients with multiple sclerosis who experience a second clinical relapse within the first two years of diagnosis?	Google Gemini	Prognosis
34	For postmenopausal women with osteopenia, does daily supplementation with vitamin D and calcium reduce the risk of fractures more effectively than exercise alone?	Microsoft Copilot	Treatment
35	For children diagnosed with ADHD, what is the link between maternal smoking during pregnancy and the occurrence of ADHD compared to children whose mothers did not smoke during pregnancy?	Microsoft Copilot	Etiology
36	Among patients with newly diagnosed rheumatoid arthritis, how do anti-cyclic citrullinated peptide (anti-CCP) antibody levels predict the likelihood of joint damage progression compared to patients without elevated anti-CCP antibody levels?	Microsoft Copilot	Prognosis
37	In elderly patients with symptomatic knee osteoarthritis, does hyaluronic acid injection improve pain and joint function more effectively than standard oral acetaminophen?	ChatGPT	Treatment
38	In adolescents, does frequent consumption of high-sugar beverages, compared to minimal or no intake, increase the risk of developing non-alcoholic fatty liver disease?	ChatGPT	Etiology
39	In patients with early-stage Alzheimer's disease, how does the presence of significant hippocampal atrophy on MRI compared to minimal atrophy influence the rate of cognitive decline over 5 years?	ChatGPT	Disease Prognosis
40	In patients with chronic obstructive pulmonary disease (COPD), does the addition of long-acting muscarinic antagonist (LAMA) to inhaled corticosteroids improve lung function, quality of life, and reduce exacerbations compared to inhaled corticosteroids alone?	Google Gemini	Treatment
41	In adults with obesity, does a diet high in processed foods increase the risk of developing type 2 diabetes mellitus?	Google Gemini	Etiology

42	How does the presence of a family history of Alzheimer's disease impact the risk of developing dementia in individuals with mild cognitive impairment?	Google Gemini	Prognosis
43	In children with acute otitis media, does the use of antibiotic therapy shorten the duration of symptoms compared to watchful waiting?	Microsoft Copilot	Treatment
44	In adolescents with major depressive disorder, how does experiencing early childhood trauma compare to genetic predisposition in influencing the development of the condition?	Microsoft Copilot	Etiology
45	In patients with acute ischemic stroke, how does the presence of early ischemic changes on initial CT scans predict long-term functional outcomes compared to those without such changes?	Microsoft Copilot	Prognosis
