# Supplementary material for: Comparing Five Generative AI Chatbots’ Answers to LLM-Generated Clinical Questions with Medical Information Scientists’ Evidence Summaries
Source: medRxiv. 2025 Sep 27:2025.09.24.25336199. Preprint. [Version 1] doi: 10.1101/2025.09.24.25336199 (PMC12486027; doi:10.1101/2025.09.24.25336199)
Supplement: Supplement 4 [file media-4.pdf]

Appendix D. Prompt for Submitting the Questions to the LLMs

#CONTEXT#
I am a medical librarian at a major academic health sciences center. In my team, our members provide evidence-based filtered summaries of the biomedical literature for use in patient care.

#OBJECTIVE#
Your task is to provide a summary of evidence that answers a clinical question I will provide to you. This involves scanning both the published and grey literature. The aim is to create a narrative statement that answers the question. When possible, the narrative statement should comment on the strengths and weaknesses of the evidence. Only use information that was available prior to [date of packet].

#STYLE#
Write in an objective, professional, and educational style in the role of a medical librarian. Write the response in a style that is directed towards medical professionals interested in understanding the available evidence.

#TONE#
Maintain a balanced and objective tone throughout the summary.

#AUDIENCE#
The target audience is clinicians providing patient care. Assume a readership that has direct experience in taking care of patients.

#RESPONSE FORMAT#
Provide an easy-to-follow narrative summary in paragraph format.

#START ANALYSIS#
If you understand, ask me to enter the clinical question.

Note: This prompt was reused from our team's previous study [1] and developed based on the COSTAR framework [2].

References:

1. Blasingame MN, Koonce TY, Williams AM, Giuse DA, Su J, Krump PA, Giuse NB. Evaluating a large language model's ability to answer clinicians' requests for evidence summaries. J Med Libr Assoc. 2025 Jan 14;113(1):65-77. DOI: https://doi.org/10.5195/jmla.2025.1985.

2. GovTech Data Science & AI Division. Prompt engineering playbook (Beta v3) [Internet]. Singapore Government Developer Portal. 2023 [cited 24 Sept 2025]. <https://www.developer.tech.gov.sg/products/collections/data-science-and-artificial-intelligence/playbooks/prompt-engineering-playbook-beta-v3.pdf>.
