# Supplementary material for: Comparing Five Generative AI Chatbots’ Answers to LLM-Generated Clinical Questions with Medical Information Scientists’ Evidence Summaries
Source: medRxiv. 2025 Sep 27:2025.09.24.25336199. Preprint. [Version 1] doi: 10.1101/2025.09.24.25336199 (PMC12486027; doi:10.1101/2025.09.24.25336199)
Supplement: Supplement 5 [file media-5.pdf]

Appendix E. Prompt for Obtaining the Key Elements

#CONTEXT#
Information scientist with medical librarianship training answering a clinical question.

#OBJECTIVE#
I will provide a clinical question and its answer. Your task is to extract only the key elements from the answer.

#STYLE#
Write in an objective, professional, and educational style in the role of a medical librarian. Write the response in a style that is directed towards medical professionals interested in understanding the available evidence.

#TONE#
Maintain a balanced and objective tone.

#AUDIENCE#
The target audience is clinicians providing patient care. Assume a readership that has direct experience in taking care of patients.

#RESPONSE FORMAT#
A numbered list can be used when more than one key element is identified.

#START ANALYSIS#
If you understand, ask me to enter the clinical question and narrative response.

In cases where the submitted narrative response was longer than the chatbot's word limit, the following text was added to the end of the prompt:

There will be two parts to the response. Please do not provide the elements until both parts are provided.

Note: All prompts were adapted from a prompt used in our team's previous study [1], which was developed based on the COSTAR framework [2].

References:

1. Blasingame MN, Koonce TY, Williams AM, Giuse DA, Su J, Krump PA, Giuse NB. Evaluating a large language model's ability to answer clinicians' requests for evidence summaries. J Med Libr Assoc. 2025 Jan 14;113(1):65-77. DOI: https://doi.org/10.5195/jmla.2025.1985.
2. GovTech Data Science & AI Division. Prompt engineering playbook (Beta v3) [Internet]. Singapore Government Developer Portal. 2023 [cited 24 Sept 2025]. <https://www.developer.tech.gov.sg/products/collections/data-science-and-artificial-intelligence/playbooks/prompt-engineering-playbook-beta-v3.pdf>.
